# The mRNA m^6^A reader YTHDF2 suppresses proinflammatory pathways and sustains hematopoietic stem cell function

**DOI:** 10.1084/jem.20200829

**Published:** 2020-11-06

**Authors:** Christopher Mapperley, Louie N. van de Lagemaat, Hannah Lawson, Andrea Tavosanis, Jasmin Paris, Joana Campos, David Wotherspoon, Jozef Durko, Annika Sarapuu, Junho Choe, Ivayla Ivanova, Daniela S. Krause, Alex von Kriegsheim, Christian Much, Marcos Morgan, Richard I. Gregory, Adam J. Mead, Dónal O’Carroll, Kamil R. Kranc

**Affiliations:** 1 Centre for Regenerative Medicine, University of Edinburgh, Edinburgh, UK; 2 Laboratory of Haematopoietic Stem Cell and Leukaemia Biology, Centre for Haemato-Oncology, Barts Cancer Institute, Queen Mary University of London, London, UK; 3 Stem Cell Program, Division of Hematology/Oncology, Boston Children’s Hospital, Boston, MA; 4 Department of Biological Chemistry and Molecular Pharmacology, Harvard Medical School, Boston, MA; 5 Georg-Speyer-Haus and Goethe University, Frankfurt, Germany; 6 Edinburgh Cancer Research UK Centre, Institute of Genetics and Molecular Medicine, Edinburgh, UK; 7 Medical Research Council Weatherall Institute of Molecular Medicine, John Radcliffe Hospital, Headington, Oxford, UK; 8 Institute for Stem Cell Research, School of Biological Sciences, University of Edinburgh, Edinburgh, UK; 9 Wellcome Centre for Cell Biology, School of Biological Sciences, University of Edinburgh, Edinburgh, UK

## Abstract

The mRNA *N*^6^-methyladenosine (m^6^A) modification has emerged as an essential regulator of normal and malignant hematopoiesis. Inactivation of the m^6^A mRNA reader YTHDF2, which recognizes m^6^A-modified transcripts to promote m^6^A-mRNA degradation, results in hematopoietic stem cell (HSC) expansion and compromises acute myeloid leukemia. Here we investigate the long-term impact of YTHDF2 deletion on HSC maintenance and multilineage hematopoiesis. We demonstrate that *Ythdf2*-deficient HSCs from young mice fail upon serial transplantation, display increased abundance of multiple m^6^A-modified inflammation-related transcripts, and chronically activate proinflammatory pathways. Consistent with the detrimental consequences of chronic activation of inflammatory pathways in HSCs, hematopoiesis-specific *Ythdf2* deficiency results in a progressive myeloid bias, loss of lymphoid potential, HSC expansion, and failure of aged *Ythdf2*-deficient HSCs to reconstitute multilineage hematopoiesis. Experimentally induced inflammation increases YTHDF2 expression, and YTHDF2 is required to protect HSCs from this insult. Thus, our study positions YTHDF2 as a repressor of inflammatory pathways in HSCs and highlights the significance of m^6^A in long-term HSC maintenance.

## Teaser

The m^6^A reader YTHDF2 is a novel regulator of inflammatory pathways in HSCs. YTHDF2 loss upregulates m^6^A-modified proinflammatory transcripts, resulting in chronic HSC inflammatory activation, myeloid bias, and functional exhaustion.

## Introduction

Emerging evidence indicates the importance of the mRNA *N*^6^-methyladenosine (m^6^A) modification in hematopoietic stem cell (HSC) biology and leukemic transformation (Vu et al., 2019). m^6^A is the most abundant internal mRNA modification, which is cotranscriptionally installed by the “m^6^A writer” complex, consisting of the METTL3/METTL14 enzymatic core and their regulator WT-associated protein (Frye et al., 2018). The modification can be reversed by m^6^A demethylases (FTO and AlkBH5), collectively called “m^6^A erasers.” m^6^A-modified transcripts are recognized by “m^6^A readers,” including nuclear YTHDC1 (YTH domain-containing 1) and cytoplasmic YTH domain-containing family member 1 (YTHDF1), YTHDF2, YTHDF3, and YTHDC2, which execute the outcome of m^6^A modification by promoting splicing, nuclear export, translation, or degradation. Recent studies demonstrated that loss of METTL3, METTL14, or FTO weakens acute myeloid leukemia (AML) propagation, whereas inactivation of METTL3 and METTL14 has deleterious consequences for normal HSC maintenance (Barbieri et al., 2017; Cheng et al., 2019; Li et al., 2017; Vu et al., 2017; Weng et al., 2018). Furthermore, inactivation of YTHDF2, which recognizes m^6^A-modified mRNA to mediate m^6^A-mRNA degradation (Du et al., 2016; Wang et al., 2015), compromises initiation and propagation of AML and results in HSC expansion (Li et al., 2018; Paris et al., 2019; Wang et al., 2018). However, the involvement of m^6^A or its readers in long-term HSC maintenance upon aging remains unknown. Here, we reveal that YTHDF2 functions to down-regulate m^6^A modified transcripts involved in the inflammatory response and maintains HSC integrity upon aging.

## Results and discussion

### YTHDF2-deficient HSCs activate proinflammatory pathways and lose their reconstitution capacity upon serial transplantation

**Figure 1.**
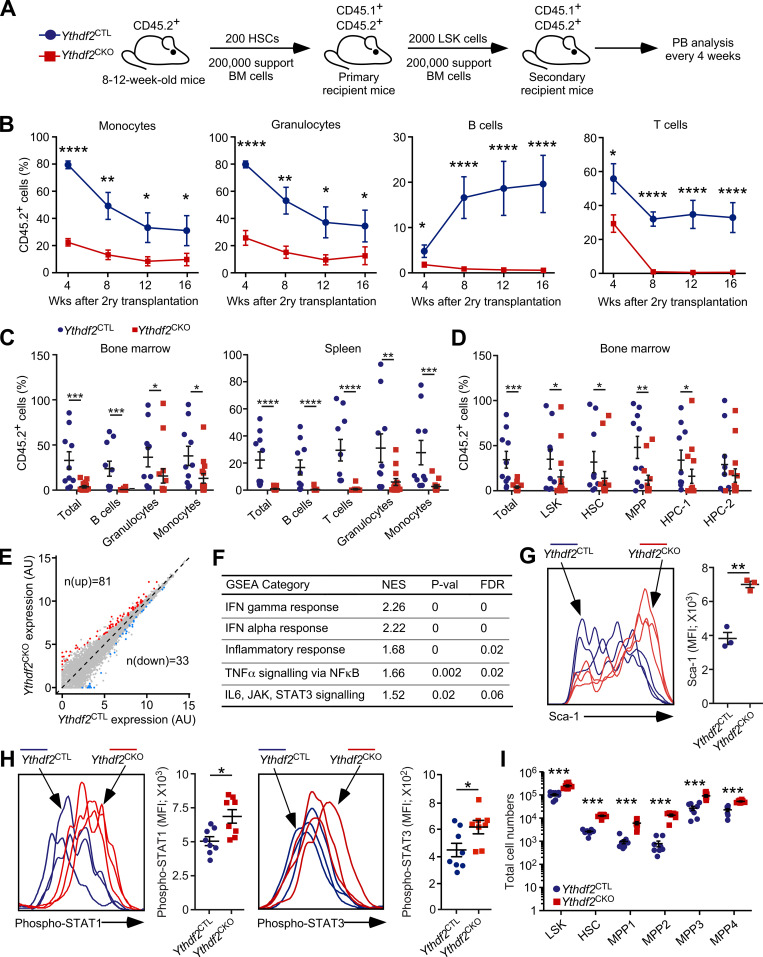
**HSCs lacking *Ythdf2* are expanded, activate proinflammatory pathways and prematurely lose function upon serial transplantation.** **(A)** Experimental design. 200 BM LSK CD48^−^CD150^+^CD45.2^+^ HSCs from 8-wk-old *Ythdf2*^CKO^ and *Ythdf2*^CTL^ mice were transplanted to lethally irradiated syngeneic CD45.1^+^/CD45.2^+^ primary recipient mice together with 2 × 10^5^ competitor CD45.1^+^ BM cells (Paris et al., 2019). After 16 wk, 2,000 CD45.2^+^LSK cells were sorted from primary recipient mice and transplanted into lethally irradiated syngeneic CD45.1^+^/CD45.2^+^ secondary recipient mice together with 2 × 10^5^ competitor CD45.1^+^ BM cells. PB of secondary recipient mice was analyzed every 4 wk, and hematopoietic compartments were analyzed 16 wk after transplantation. **(B)** Percentage of CD45.2^+^ cells in the PB CD11b^+^Gr-1^−^ monocyte, CD11b^+^Gr-1^+^ granulocyte, CD19^+^ B cell, and CD4^+^ and CD8^+^ T cell compartments of secondary recipient mice over 16 wk. Data represent mean ± SEM; *, P < 0.05; ****, P < 0.0001 (Mann–Whitney *U* test); at least two independent experiments were performed. **(C)** Percentage of CD45.2^+^ cells in the BM and spleen overall and in the monocyte, granulocyte, B cell, and T cell compartments of secondary recipient mice 16 wk after transplantation. Data represent mean ± SEM; *, P < 0.05; **, P < 0.01; ***, P < 0.001; ****, P < 0.0001 (Mann–Whitney *U* test); at least two independent experiments were performed. **(D)** Percentage of CD45.2^+^ cells in the BM of secondary recipients and in the LSK, LSK CD48^−^CD150^+^ HSC, LSK CD48^−^CD150^−^ MPP, LSK CD48^+^CD150^−^ HPC-1, and LSK CD48^+^CD150^+^ HPC-2 cell compartments of secondary recipient mice 16 wk after transplantation. Data represent mean ± SEM; *, P < 0.05; **, P < 0.01; ***, P < 0.001 (Mann–Whitney *U* test); at least two independent experiments were performed. **(E)** Transcript expression scatterplot from *Ythdf2*^CKO^ and *Ythdf2*^CTL^ HSCs from 8–12-wk-old mice (*n* = 4). Significantly dysregulated transcripts are highlighted and counted (FDR < 0.05; log_2_ fold change < 1.2). **(F)** GSEA showing up-regulated pathways in HSCs from 8–12-wk-old *Ythdf2*^CKO^ mice compared with HSCs from *Ythdf2*^CTL^ mice. **(G)** Histogram and median Sca-1 fluorescence intensity in LSK CD48^−^CD150^+^ HSCs from *Ythdf2*^CKO^ and *Ythdf2*^CTL^ mice determined by flow cytometry. Data represent mean ± SEM; **, P < 0.01 (Mann–Whitney *U* test); at least two independent experiments were performed. **(H)** Phospho-STAT1 and phospho-STAT3 levels in LSK cells determined by flow cytometry. Data represent mean ± SEM; *, P < 0.05 (Mann–Whitney *U* test); at least two independent experiments were performed. **(I)** Total numbers of LSK cells, HSCs (LSK CD34^−^CD135^−^CD150^+^CD48^−^), and MPP1 (also referred to as short-term HSCs; LSK CD34^+^CD135^−^CD150^+^CD48^−^), MPP2 (LSK CD34^+^CD135^−^CD150^+^CD48^+^), MPP3 (LSK CD34^+^CD135^−^CD150^−^CD48^+^), and MPP4 (LSK CD34^+^CD135^+^CD150^−^CD48^+^) cell populations in BM of 8–12-wk-old *Ythdf2*^CKO^ and *Ythdf2*^CTL^ mice. Data represent mean ± SEM; ***, P < 0.001 (Mann–Whitney *U* test); at least two independent experiments were performed. AU, arbitrary units; MFI, mean fluorescence intensity.

**Figure S1.**
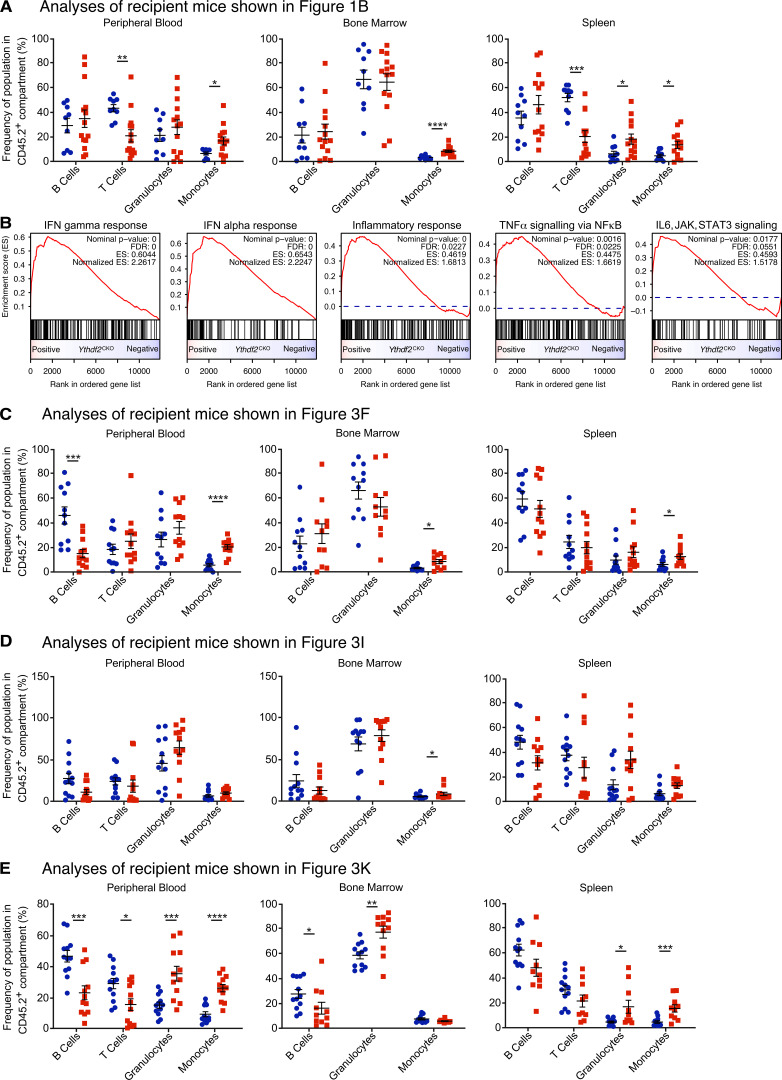
**Molecular and cellular features of *Ythdf2-*deficient HSCs.** **(A)** BM LSK CD48^−^CD150^+^CD45.2^+^ HSCs from 8-wk-old *Ythdf2*^CKO^ and *Ythdf2*^CTL^ mice were transplanted to lethally irradiated syngeneic CD45.1^+^/CD45.2^+^ primary recipient mice together with 2 × 10^5^ competitor CD45.1^+^ BM cells (as published in Paris et al., 2019). After 16 wk, 2,000 CD45.2^+^ LSK cells were sorted from primary recipient mice and transplanted into lethally irradiated syngeneic CD45.1^+^/CD45.2^+^ secondary recipient mice together with 2 × 10^5^ competitor CD45.1^+^ BM cells (as described in Fig. 1 A). The frequency of CD19^+^ B cells, CD4^+^ or CD8^+^ T cells, CD11b^+^Gr-1^+^ granulocytes, and CD11b^+^Gr-1^−^ monocytes in the CD45.2^+^ compartment of PB, BM, and spleen of secondary transplanted mice was analyzed 16 wk after transplantation. Analysis of the percentage of CD45.2^+^ cells in each compartment is presented in Fig. 1, B and C (*n* = 12). Data represent mean ± SEM; *, P < 0.05; **, P < 0.01; ***, P < 0.001; ****, P < 0.0001 (Mann–Whitney *U* test); at least two independent experiments were performed. **(B)** HSCs lacking *Ythdf2* display proinflammatory gene signatures. GSEA of differential gene expression in *Ythdf2*^CKO^ versus *Ythdf2*^CTL^ HSCs from young animals. Results for five significant (FDR < 0.1) inflammation-related categories are shown (Hallmark, MSigDB v6.2). Genes were ordered by log P value but ranked in the same sense as log fold-change. **(C)** *Ythdf2*^CKO^ and control *Ythdf2*^CTL^ mice were aged for 1 yr before sorting and transplantation of 200 BM LSK CD48^−^CD150^+^CD45.2^+^ HSCs into lethally irradiated 8–10-wk-old syngeneic CD45.1^+^/CD45.2^+^ recipient mice (*n =* 12) together with 2 × 10^5^ CD45.1^+^ competitor BM cells (as described in Fig. 3 A). After 16 wk, the frequency of B cells, T cells, granulocytes, and monocytes in the CD45.2^+^ compartment of PB, BM, and spleen of recipient mice was analyzed. Analysis of the percentage of CD45.2^+^ cells within lineages and stem cell compartments is presented in Fig. 3, F and G. Data represent mean ± SEM; *, P < 0.05; ***, P < 0.001; ****, P < 0.0001 (Mann–Whitney *U* test); at least two independent experiments were performed. **(D and E)** 8-wk-old *Ythdf2*^fl/fl^;*Mx1-Cre* (*Ythdf2*^iCKO^) and control *Ythdf2*^fl/fl^ (*Ythdf2*^CTL^) mice were treated with six sequential doses of plpC (every other day) to induce deletion of *Ythdf2.* BM LSK CD48^−^CD150^+^ CD45.2^+^ HSCs were sorted from 34-wk-old (D) and 60-wk-old (E) *Ythdf2*^CTL^ and *Ythdf2*^iCKO^ mice (as described in Fig. 3 H) before transplantation into lethally irradiated syngeneic CD45.1^+^/CD45.2^+^ recipient mice together with 2 × 10^5^ competitor CD45.1^+^ BM cells. The frequency of B cells, T cells, granulocytes, and monocytes in the CD45.2^+^ compartment of PB, BM, and spleen of recipient mice was analyzed 16 wk after transplantation. Analysis of the percentage of CD45.2^+^ cells in lineage and stem cell compartments is presented in Fig. 3, I–L (*n* = 12). Data represents mean ± SEM; *, P < 0.05; **, P < 0.01; ***, P < 0.001; ****, P < 0.0001 (Mann–Whitney *U* test); at least two independent experiments were performed.

We have recently demonstrated that hematopoiesis-specific *Vav-iCre*–mediated deletion of *Ythdf2* results in HSC expansion and compromises AML initiation and propagation (Paris et al., 2019). We used the conditional and reporter *Ythdf2*^fl^ mouse allele in which exon 2 of *Ythdf2* was flanked by *loxP* sites and GFP was introduced after the start codon of *Ythdf2*, generating a fully functional GFP-YTHDF2 fusion protein (Ivanova et al., 2017). Our published data indicated that 8–12-wk-old *Ythdf2*^fl/fl^;*Vav-iCre* (conditional knockout, *Ythdf2*^CKO^) mice displayed largely unaffected hematopoiesis compared with control *Ythdf2*^fl/fl^ (control, *Ythdf2*^CTL^) mice, with modest decreases in numbers of white and red blood cells, B cells, and CD8^+^ T cells (Paris et al., 2019). Furthermore, while HSCs from 8–12-wk-old *Ythdf2*^CKO^ mice displayed myeloid bias upon primary transplantation and robustly contribute to the bone marrow (BM) HSC and progenitor cell compartments (Paris et al., 2019), the role of YTDHF2 in long-term HSC maintenance remains unknown. To address this, we performed secondary transplantation assays by sorting CD45.2^+^ Lin^−^c-Kit^+^Sca-1^+^ (LSK) cells from primary recipients of *Ythdf2*^CKO^ and *Ythdf2*^CTL^ HSCs (Paris et al., 2019) and retransplanting them (together with supporting BM cells) into lethally irradiated secondary recipient mice (Fig. 1 A). Strikingly, *Ythdf2*^CKO^ HSCs failed to efficiently repopulate long-term multilineage hematopoiesis and stem and progenitor cell compartments in secondary recipients (Fig. 1, B–D) and displayed myeloid bias (Fig. S1 A). Thus, HSCs critically require YTHDF2 to sustain their function upon serial transplantation.

To understand the cause of the failure of *Ythdf2*-deficient HSCs upon serial transplantation, we next set out to determine the molecular signature of *Ythdf2*-deficient HSCs. *Ythdf2* deficiency resulted in deregulated gene expression (Fig. 1 E), with 81 up-regulated transcripts and 33 down-regulated transcripts (false discovery rate [FDR] < 0.05) in *Ythdf2*^CKO^ compared with *Ythdf2*^CTL^ HSCs. Strikingly, gene set enrichment analysis (GSEA) on *Ythdf2*^CKO^ versus *Ythdf2*^CTL^ HSCs revealed significant up-regulation of numerous inflammation-related processes, such as IFN-α response, IFN-γ response, inflammatory response, TNF-α signaling, and IL6/JAK/STAT3 signaling (Fig. 1 F and Fig. S1 B).

Given that proinflammatory signals are known to drive overproliferation of HSCs, ultimately resulting in loss of HSC function (Essers et al., 2009; Pietras, 2017), we aimed to confirm the proinflammatory signature in *Ythdf2*-deficient HSCs using additional approaches. We found that HSCs from *Ythdf2*^CKO^ mice displayed increased expression of cell surface Sca-1 protein (Fig. 1 G), which is used as a proxy of IFN signaling (Essers et al., 2009). Furthermore, *Ythdf2*-deficient HSCs showed elevated levels of phosphorylated forms of STAT1 and STAT3 proteins, key mediators of IFN signaling (Fig. 1 H).

Given that *Ythdf2-*deficient HSCs showed up-regulation of proinflammatory pathways and displayed a myeloid bias (a hallmark of inflammation), we aimed to determine whether the primitive progenitor compartment also reflected innate immune activation in *Ythdf2*^CKO^ mice. During steady-state normal hematopoiesis, HSCs generate functionally distinct lineage-biased multipotent progenitors (MPPs), with MPP2/3 and MPP4 populations predominantly sustaining myeloid and lymphoid lineages, respectively (Pietras et al., 2015). Stressful conditions such as infection, stress, or injury induce proinflammatory stimuli, which not only activate HSCs but also cause expansion of MPPs, in particular myeloid-biased MPP2/3 populations, thereby driving myeloid differentiation at the expense of HSC integrity (Essers et al., 2009; Pietras et al., 2016; Yamashita and Passegue, 2019; Zhang et al., 2017; Zhang et al., 2016). Consistent with the activation of proinflammatory pathways, we found that 8–12-wk-old *Ythdf2*^CKO^ mice displayed expansion of HSCs and a concurrent increase in all four main subpopulations, namely MPP1-4 cells (Fig. 1 I). Thus, hematopoietic-specific deletion of *Ythdf2* results in a constitutive up-regulation of the proinflammatory signature, expansion of HSCs and MPPs, and a failure of HSC function upon serial transplantation.

### YTHDF2 functions to suppress m^6^A-modified proinflammatory transcripts

**Figure 2.**
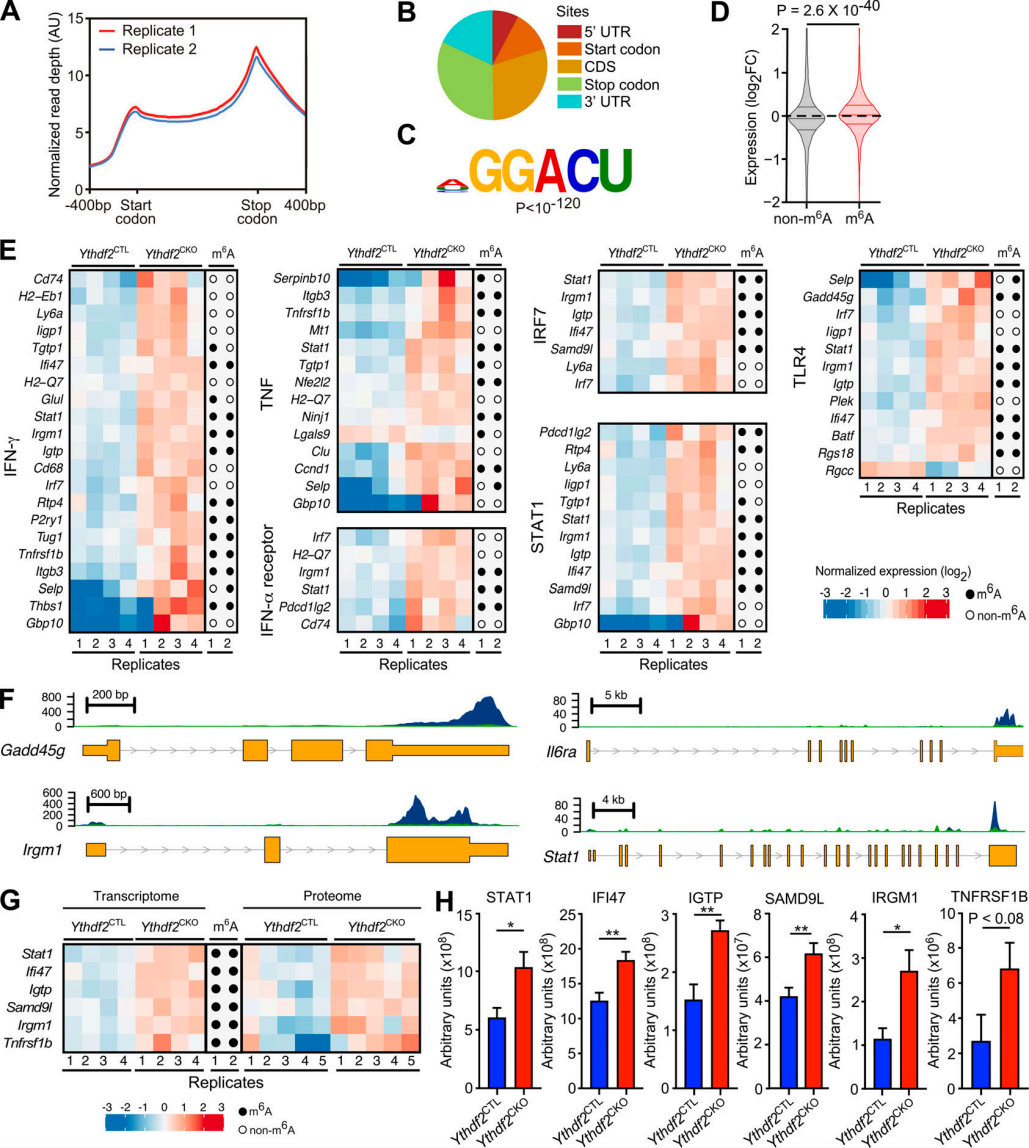
***Ythdf2-*deficient HSCs display up-regulation of m^6^A-modified transcripts involved in multiple proinflammatory pathways.** **(A)** m^6^A-seq read depth along a transcript body model for two replicate c-Kit^+^ *Ythdf2*^CTL^ cell samples. Transcripts models were extended 400 nucleotides upstream and downstream of the start and stop codons, respectively. **(B)** Location distribution of m^6^A peaks in gene regions. m^6^A peak overlaps with different transcript regions (5′ UTR; start codon; coding sequence, CDS; stop codon; 3′ UTR). **(C)** Enriched m^6^A motif found by Homer in peaks detected by macs2 v2.1.1.20160309. **(D)** Violin plot of expression change between *Ythdf2*^CTL^ and *Ythdf2*^CKO^ BM LSK CD48^−^CD150^+^ HSCs (*n* = 4) from 8–12-wk-old mice for nonmethylated (nonm^6^A) and methylated (m^6^A) transcripts determined by m^6^A-seq in c-Kit^+^ cells. **(E)** Heatmaps of genes representing IPA-defined IFN-γ, TNF-α, IFN-α, IFN regulatory factor 7, STAT1 and TLR4 signaling pathways in *Ythdf2*^CKO^ compared with *Ythdf2*^CTL^ HSCs from 8–12-wk-old mice (*n* = 4). Transcript methylation peak presence in two replicates from c-Kit^+^ cells depicted to the right by filled (m^6^A-modified) or unfilled (no m^6^A modification detected) circles. **(F)** m^6^A signal in key transcripts involved in inflammatory responses. Read depth from two replicates of m^6^A immunoprecipitation (blue) and RNA-seq input (green). Transcript regions are depicted as exonic (yellow) and intronic (gray). **(G and H)** Methylated proinflammatory transcripts up-regulated in *Ythdf2*^CKO^ HSCs, which also show increased protein abundance in immortalized *Ythdf2*^CKO^ c-Kit^+^ cells (Paris et al., 2019). **(G)** Heatmap to left represents transcript abundance in HSC samples. Transcript m^6^A modification in c-Kit^+^ cells is indicated in the center. Heatmap to right represents protein abundance in c-Kit^+^ cells. Heatmap colors represent expression z-scores for a given row. **(H)** Peptide spectral counts (expressed as arbitrary units) from immortalized *Ythdf2*^CTL^ and *Ythdf2*^CKO^ c-Kit^+^ cells. Data represent mean ± SEM; *n* = 5 per genotype; *, P < 0.05; **, P < 0.01 (Mann–Whitney *U* test). AU, arbitrary units.

**Figure S2.**
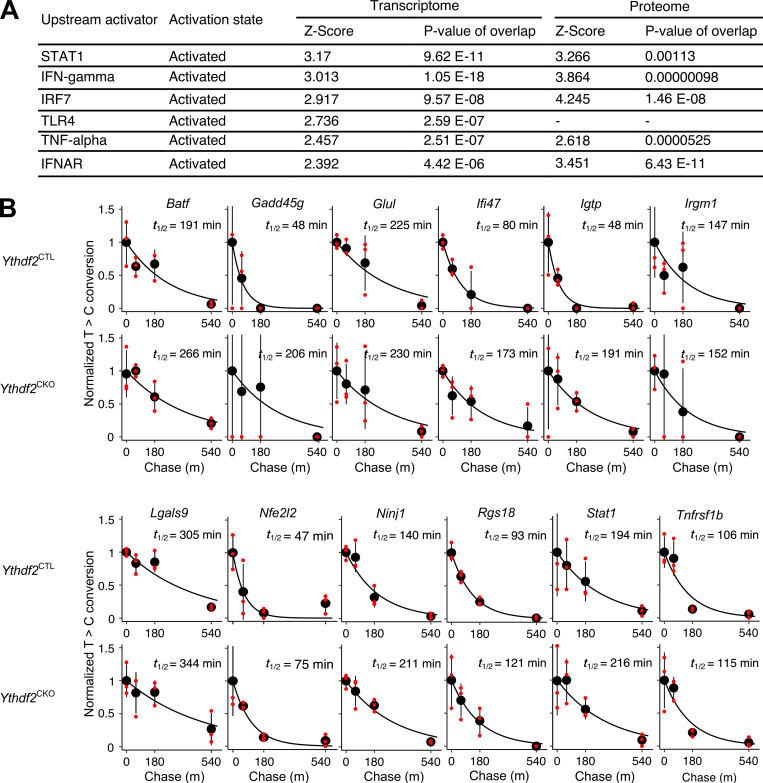
**Molecular properties of HSCs lacking *Ythdf2*.** **(A)** IPA of RNA-seq and mass spectroscopy from *Ythdf2*^CTL^ and *Ythdf2*^CKO^ HSCs or c-Kit^+^ cells, respectively. Z-score represents the degree of activation of the pathway, with a threshold for significance set at 2. P value shown refers to the overlap between target genes or proteins in the dataset and target genes in the IPA database. RNA-seq, *n* = 4; proteomics, *n* = 5. **(B)** Decay curves for transcripts in *Ythdf2*^CTL^ (upper panels) and *Ythdf2*^CKO^ (lower panels) c-Kit^+^ cells are shown. The center value and the error bars at each time point indicate the conversion rate mean and SD, respectively. Conversion rates for each biological replicate are indicated with dots. The half-life for each graph is also shown.

YTHDF2 regulates the transcriptome by binding m^6^A modified transcripts to promote their decay through deadenylation (Du et al., 2016; Wang et al., 2014). To identify YTHDF2 target transcripts, we aimed to determine mRNAs that were methylated in normal conditions and up-regulated in *Ythdf2*^CKO^ HSCs. m^6^A sequencing (m^6^A-seq) in c-Kit^+^ hematopoietic stem and progenitor cells was used to determine transcriptome-wide m^6^A distribution. This identified the expected distribution of m^6^A within the transcriptome (i.e., enrichment around the stop codons; Fig. 2, A and B) as well as the m^6^A consensus motif (DRACH; D = A, G, or U; R = purine; and H = A, C, or U; Fig. 2 C). Consistent with the role of YTHDF2 in promoting degradation of m^6^A-modified transcripts, we found that transcripts that contain m^6^A modification (P = 2.6 × 10^−40^, 8,253 genes) show increased expression in *Ythdf2*^CKO^ HSCs (Fig. 2 D). Ingenuity pathway analysis (IPA) indicated that up-regulated genes are enriched for transcripts related to inflammatory responses (including IFN-γ, TNF-α, IFN-α, IFN regulatory factor 7, STAT1, and TLR4; Fig. 2 E and Fig. S2 A). Importantly, m^6^A-seq revealed that a large proportion of these key up-regulated transcripts in HSCs are m^6^A modified, including key genes involved in inflammation such as *Stat1*, *Il6ra*, and *Gadd45g* (Fig. 2, E and F).

The up-regulation of m^6^A-modified transcripts involved in inflammation upon loss of YTHDF2 may be a consequence of an increase in their half-life. Given that the measurement of transcript half-life in HSCs is technically not possible, to address this, we analyzed our SLAM-seq dataset from immortalized c-Kit^+^ cells derived from *Ythdf2*^CTL^ and *Ythdf2*^CKO^ mice (Paris et al., 2019), which allowed us to measure the transcriptome-wide mRNA half-life (Herzog et al., 2017). We found that multiple m^6^A-modified transcripts that are up-regulated upon *Ythdf2* deletion (Fig. 2 E) also have their half-lives increased in the absence of *Ythdf2* (Fig. S2 B).

Finally, we next asked whether up-regulation of m^6^A-modified transcripts upon *Ythdf2* deletion corresponds to an increase in their protein level. Mass spectrometry can be used to obtain the comprehensive but incomplete quantitative measurement of the proteome. IPA of quantitative mass spectrometry–based proteomics of immortalized *Ythdf2*^CTL^ and *Ythdf2*^CKO^ c-Kit^+^ cells indicated that up-regulated proteins are related to the same inflammatory responses found in the transcriptomic data (Fig. S2 A). Strikingly, based on the coverage of the proteomics dataset, we found that protein level of STAT1, IFN-γ inducible protein 47, IFN-γ–induced GTPaseP, sterile α motif domain containing 9 like, immunity-related GTPase family M protein 1, and TNF receptor 2, whose corresponding transcripts were up-regulated and methylated upon *Ythdf2* deletion, were elevated in *Ythdf2*-deficient cells (Fig. 2, G and H). Thus, YTHDF2 suppresses m^6^A-modified mRNAs, which are involved in multiple proinflammatory pathways within HSCs. Given that *Ythdf2* deletion results in up-regulation of multiple m^6^A-modified transcripts in HSCs, future genetic rescue experiments will reveal their relative contributions to their failure upon serial transplantation.

### Aged HSCs lacking *Ythdf2* undergo expansion and sustain steady-state hematopoiesis but fail to reconstitute multilineage hematopoiesis upon transplantation

**Figure S3.**
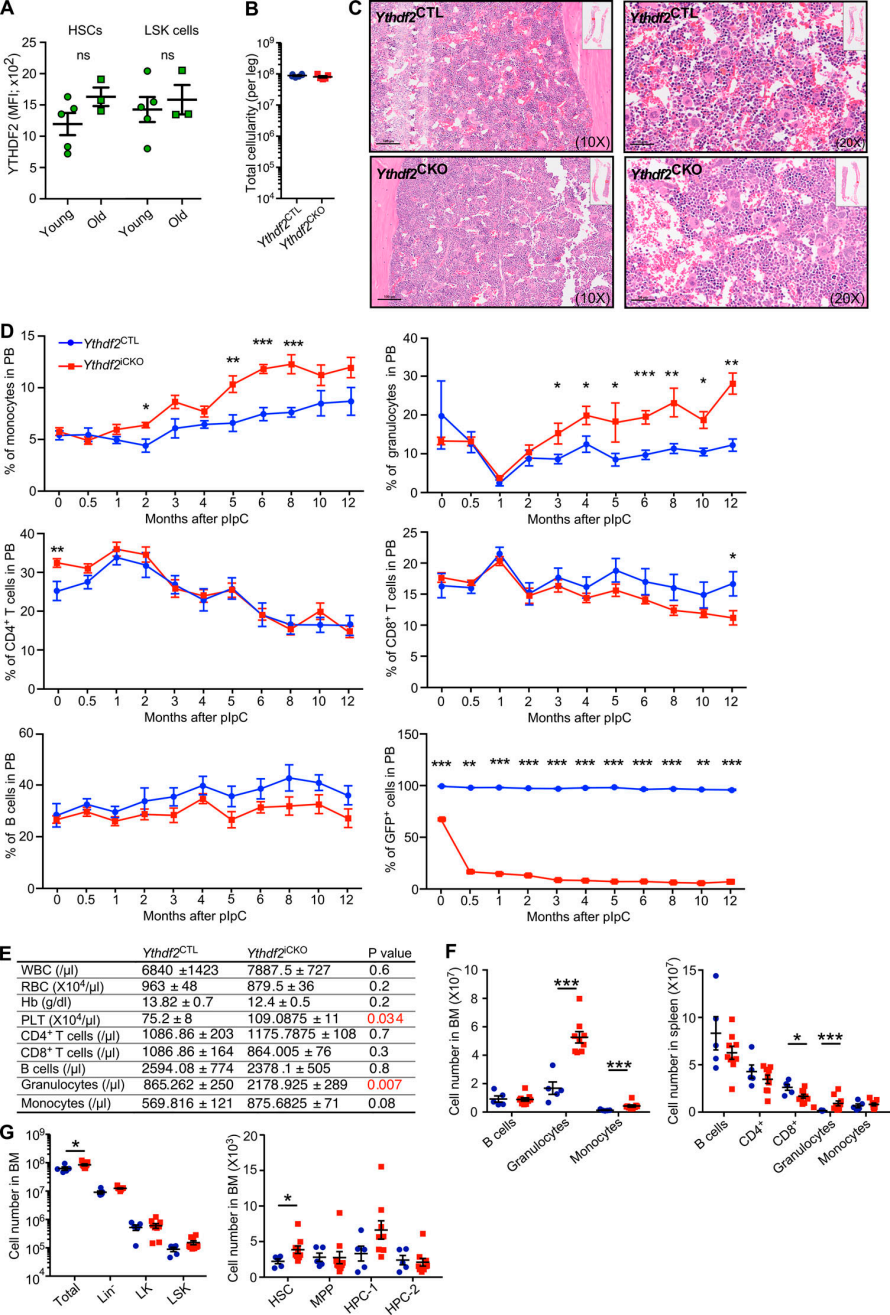
**Inducible deletion of *Ythdf2* from adult mice does not derail long-term steady-state hematopoiesis but results in a progressive myeloid bias and HSC failure upon aging.** **(A)** Modal YTHDF2-GFP fluorescence assayed by flow cytometry in LSKs and LSK CD48^−^CD150^+^ HSCs from 8–12-wk-old and 60–65-wk-old *Ythdf2*^CTL^ mice. Data represent mean ± SEM; ns, not significant (Mann–Whitney *U* test); at least two independent experiments were performed. **(B)** Total BM cellularity of one leg from 60–65-wk-old *Ythdf2*^CTL^ and *Ythdf2*^CKO^ mice (*n* ≥ 5). Data represent mean ± SEM; at least two independent experiments were performed. **(C)** Representative sections of H&E-stained *Ythdf2*^CTL^ and *Ythdf2*^CKO^ BM at 10× and 20× magnification (*n* ≥ 5). **(D)** Percentage of CD19^+^B220^+^ B cells, CD4^+^ T cells, CD8^+^ T cells, CD11b^+^Gr-1^+^ granulocytes, CD11b^+^Gr-1^−^ monocytes, and GFP-YTHDF2^+^ cells in PB of *Ythdf2*^iCKO^ and *Ythdf2*^CTL^ mice over 12 mo after pIpC treatment. Bottom right, loss of expression of GFP-YTHDF2 following pIpC treatment (*n* ≥ 5). Data represents mean ± SEM; *, P < 0.05; **, P < 0.01; ***, P < 0.001 (Mann–Whitney *U* test); at least two independent experiments were performed. **(E)** PB counts of *Ythdf2*^CTL^ and Ythdf2^iCKO^ mice 12 mo after pIpC treatment (*n* = 5–8). Data represent mean ± SEM; P value by Mann–Whitney *U* test; at least two independent experiments were performed. **(F)** Total number of differentiated cell populations in BM and spleens 12 mo after pIpC treatment (*n* = 5–9). Data represent mean ± SEM; *, P < 0.05; ***, P < 0.001 (Mann–Whitney *U* test); at least two independent experiments were performed. **(G)** Total cell numbers of BM primitive cell populations. HSCs, LSK CD48^−^CD150^+^ HSCs; MPP, LSK CD48^−^CD150^−^; HPC-1, LSK CD48^+^CD150^−^, HPC-2 LSK CD48^+^CD150^+^, and LK Lin^−^Sca-1^−^c-Kit^+^ myeloid progenitors 12 mo after pIpC treatment (*n* = 5–9). Data represent mean ± SEM, *, P < 0.05 (Mann–Whitney *U* test); at least two independent experiments were performed.

**Figure 3.**
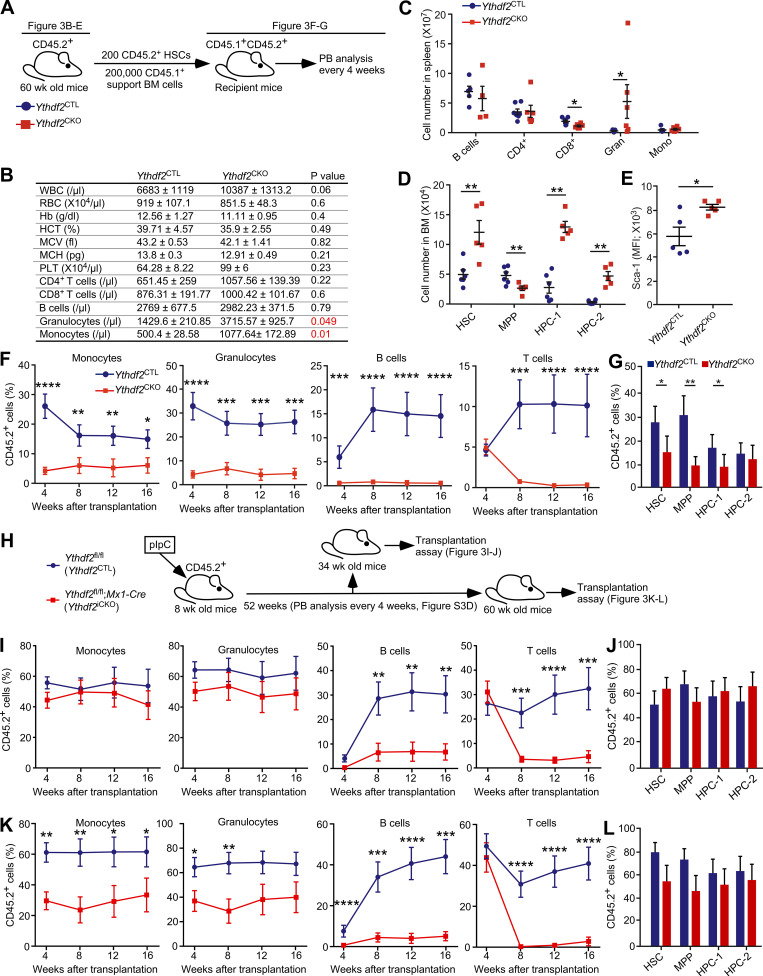
**HSCs lacking *Ythdf2* display a progressive myeloid bias and loss of stem cell activity upon aging.** **(A)** *Ythdf2*^fl/fl^;*Vav-iCre* (*Ythdf2*^CKO^) and control *Ythdf2*^fl/fl^ (*Ythdf2*^CTL^) mice were aged for 1 yr, followed by analyses of their steady-state hematopoiesis (B–E). Furthermore, 200 BM LSK CD48^−^CD150^+^CD45.2^+^ HSCs were sorted and transplanted into lethally irradiated 8–10-wk-old syngeneic CD45.1^+^/CD45.2^+^ recipient mice (*n =* 12 per genotype) together with 2 × 10^5^ CD45.1^+^ competitor BM cells (F and G). **(B)** PB counts of 60–65-wk-old *Ythdf2*^CKO^ and *Ythdf2*^CTL^ mice (*n* ≥ 5); P < 0.05 is highlighted in red. Data represent mean ± SEM; at least two independent experiments were performed. **(C)** Total number of CD19^+^B220^+^ B cells, CD4^+^ T cells, CD8^+^ T cells, CD11b^+^Gr-1^+^ granulocytes, and CD11b^+^Gr-1^−^ monocytes in spleens of 60–65-wk old *Ythdf2*^CKO^ and *Ythdf2*^CTL^ mice (*n* ≥ 5). Data represent mean ± SEM; *, P < 0.05 (Mann–Whitney *U* test); at least two independent experiments were performed. **(D)** Total number of HSCs, LSK CD48^−^CD150^−^ MPPs, and primitive hematopoietic progenitor cells (i.e., LSK CD48^+^CD150^−^ HPC-1 and LSK CD48^+^CD150^+^ HPC-2 populations) in BM of 60–65-wk-old *Ythdf2*^CKO^ and *Ythdf2*^CTL^ mice (*n* ≥ 5). Data represent mean ± SEM; **, P < 0.01 (Mann–Whitney *U* test); at least two independent experiments were performed. **(E)** Median Sca-1 fluorescence intensity in HSCs from 60–65-wk-old *Ythdf2*^CKO^ or *Ythdf2*^CTL^ mouse BM determined by flow cytometry (*n* ≥ 5). Data represent mean ± SEM; *, P < 0.05 (Mann–Whitney *U* test); at least two independent experiments were performed. **(F)** Percentage of CD45.2^+^ cells in the monocyte, granulocyte, B cell, and T cell compartments of recipients transplanted with HSCs from 60–65-wk-old CD45.2^+^ *Ythdf2*^CKO^ or *Ythdf2*^CTL^ mice (*n* = 12). Data represent mean ± SEM; ***, P < 0.001; ****, P < 0.0001 (Mann–Whitney *U* test); at least two independent experiments were performed. **(G)** Percentage of CD45.2^+^ cells in the BM HSC, MPP, HPC-1, and HPC-2 cell compartments of recipient mice 16 wk after transplantation (*n* = 12). Data represent mean ± SEM; *, P < 0.05; **, P < 0.01 (Mann–Whitney *U* test); at least two independent experiments were performed. **(H)** 8-wk-old *Ythdf2*^fl/fl^;*Mx1-Cre* (*Ythdf2*^iCKO^) and control *Ythdf2*^fl/fl^ (*Ythdf2*^CTL^) mice were treated with six sequential doses of plpC (given every alternate day) to induce deletion of *Ythdf2.* PB samples were taken at day 0 and over a 52-wk period after pIpC treatment (Fig. S3 D). 200 BM LSK CD48^−^CD150^+^ CD45.2^+^ HSCs sorted from 34-wk-old (Fig. 3, I and J) and 60-wk-old (Fig. 3, K and L) *Ythdf2*^CTL^ and *Ythdf2*^iCKO^ mice were transplanted into lethally irradiated syngeneic CD45.1^+^/CD45.2^+^ recipient mice together with 2 × 10^5^ competitor CD45.1^+^ BM cells (*n* = 12). **(I)** Percentage of CD45.2^+^ cells overall in the monocyte, granulocyte, B cell, and T cell compartments of lethally irradiated recipient mice transplanted with HSCs from 34-wk-old CD45.2^+^ *Ythdf2*^iCKO^ or *Ythdf2*^CTL^ mice (*n* = 12). Data represent mean ± SEM; **, P < 0.01; ***, P < 0.001; ****, P < 0.0001 (Mann–Whitney *U* test); at least two independent experiments were performed. **(J)** Percentage of CD45.2^+^ cells in the BM HSC, MPP, HPC-1, and HPC-2 cell compartments of recipient mice 16 wk after transplantation with HSCs from 34-wk-old *Ythdf2*^iCKO^ or *Ythdf2*^CTL^ mice (*n* = 12). Data represent mean ± SEM, at least two independent experiments were performed. **(K)** Percentage of CD45.2^+^ cells overall in the monocyte, granulocyte, B cell, and T cell compartments of lethally irradiated recipient mice transplanted with HSCs from 60-wk-old CD45.2^+^ *Ythdf2*^iCKO^ or *Ythdf2*^CTL^ mice (*n* = 12). Data represent mean ± SEM; *, P < 0.05; **, P < 0.01; ***, P < 0.001; ****, P < 0.0001 (Mann–Whitney *U* test); at least two independent experiments were performed. **(L)** Percentage of CD45.2^+^ cells in the BM HSC, MPP, HPC-1, and HPC-2 cell compartments of recipient mice 16 wk after transplantation with HSCs from 60-wk-old *Ythdf2*^iCKO^ or *Ythdf2*^CTL^ mice (*n* = 12). Data represent mean ± SEM; at least two independent experiments were performed.

Proinflammatory signals are known to drive overproliferation of HSCs, promote myeloid bias, and ultimately lead to the loss of HSC activity (Essers et al., 2009; Pietras, 2017). Furthermore, proinflammatory pathways are up-regulated in aged HSCs, indicating their roles in stem cell aging (Yamashita and Passegue, 2019). We found that both young and aged HSCs expressed comparable levels of the YTHDF2 protein (Fig. S3 A). To investigate the functional significance of YTHDF2 in unperturbed long-term multilineage hematopoiesis and maintenance of the HSC pool over a long period of time, we aged *Ythdf2*^CKO^ and *Ythdf2*^CTL^ mice for 1 yr (Fig. 3 A). Analyses of 60–65-wk-old *Ythdf2*^CKO^ mice revealed increased numbers of peripheral blood (PB) granulocytes and monocytes (i.e., myeloid bias); however, hematopoiesis was otherwise largely unaffected (Fig. 3, B and C; and Fig. S3, B and C). Furthermore, 60–65-wk-old *Ythdf2*^CKO^ mice displayed expansion of BM HSCs and primitive hematopoietic progenitor cell populations (Fig. 3 D). HSCs from these mice showed increased expression of Sca-1 (Fig. 3 E), indicating constitutive activation of proinflammatory signals. To test the ability of the expanded aged HSCs to reconstitute multilineage hematopoiesis, we competitively transplanted HSCs from 60–65-wk-old *Ythdf2*^CKO^ and *Ythdf2*^CTL^ mice into lethally irradiated recipients (Fig. 3 A). While HSCs from aged *Ythdf2*^CTL^ mice robustly repopulated both myeloid and lymphoid lineages, HSCs from aged *Ythdf2*^CKO^ mice failed to efficiently reconstitute short- and long-term multilineage hematopoiesis in the recipients and displayed a myeloid bias (Fig. 3 F and Fig. S1 C). The contribution of *Ythdf2*-deficient HSCs to the HSC compartment of the recipients was also decreased compared with that of control HSCs (Fig. 3 G), albeit this was not as striking as their inability to reconstitute multilineage hematopoiesis (Fig. 1 F). Thus, while long-term *Ythdf2* deficiency in the hematopoietic system does not derail steady-state multilineage hematopoiesis, it devastates the multilineage reconstitution capacity of HSCs. Given that the inflammatory pathways affect multiple HSC fates (Essers et al., 2009; Pietras, 2017; Pietras et al., 2014; Pietras et al., 2016), future investigations will be required to determine the exact impact of *Ythdf2* deletion on HSC survival, self-renewal, differentiation capacity, and homing.

### Inducible *Ythdf2* deletion results in a progressive loss of lymphoid potential, with concurrent myeloid bias and subsequent HSC failure

To delete *Ythdf2* in adult mice in an inducible manner, we generated *Ythdf2*^iCKO^ mice in which *Ythdf2* can be ablated acutely in hematopoietic cells upon administration of polyinosinic-polycytidylic acid (pIpC). *Ythdf2*^iCKO^ and *Ythdf2*^CTL^ mice were treated with pIpC and analyzed 26 and 52 wk later (Fig. 3 H). Inducible *Ythdf2* deletion resulted in increased myeloid output (Fig. S3, D–F) and HSC expansion (Fig. S3 G), but multilineage hematopoiesis was otherwise largely unaffected (Fig. S3, D–F). Transplanted HSCs from mice 26 wk after *Ythdf2* deletion reconstituted myeloid lineages, failed to efficiently reconstitute lymphoid lineages (Fig. 3 I and Fig. S1 D), and contributed equally to the HSC compartment of recipients compared with control HSCs (Fig. 3 J). Strikingly, HSCs from mice 52 wk after *Ythdf2* ablation failed to efficiently reconstitute both myeloid and lymphoid lineages and displayed a myeloid bias (Fig. 3 K and Fig. S1 E), while their ability to contribute to the HSC compartment of the recipients was largely unaffected (Fig. 3 L). Thus, inducible *Ythdf2* deletion in adult mice causes a progressive loss of lymphoid output, myeloid bias upon aging, and an eventual failure of aged HSCs to reconstitute long-term multilineage hematopoiesis.

### YTHDF2 protein expression in HSCs is induced by acute and chronic inflammation

**Figure 4.**
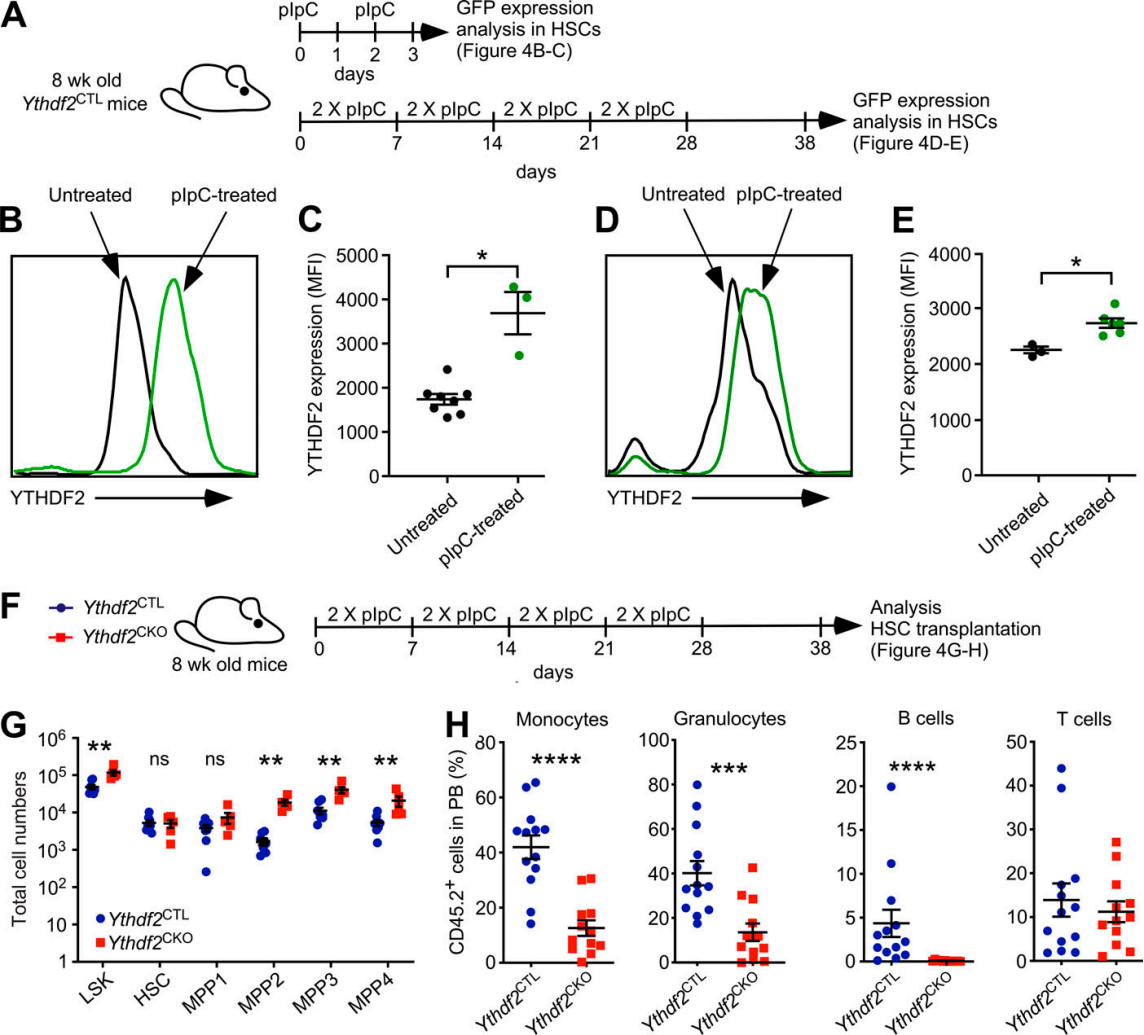
**YTHDF2 expression responds to acute and chronic inflammation.** **(A)** *Ythdf2*^fl/fl^ (*Ythdf2*^CTL^) mice were treated with two doses of plpC on days 0 and 2 before analysis on day 3 (Fig. 4, B and C) or treated with two doses of plpC per week for 4 wk and allowed to recover for 10 d before analysis (Fig. 4, D and E). Untreated littermate controls were analyzed at equal time points. **(B)** Representative histogram of modal YTHDF2-GFP fluorescence assayed by flow cytometry in LSK CD48^−^CD150^+^ HSCs from untreated littermate controls or mice treated with two doses of plpC. At least two independent experiments were performed. **(C)** Median YTHDF2-GFP fluorescence intensity (MFI) of the data presented in B (*n* = 3 and *n* = 8 biological replicates, for treated and untreated, respectively). Data represent mean ± SEM; *, P < 0.05; (Mann–Whitney *U* test); at least two independent experiments were performed. **(D)** Representative histogram of modal YTHDF2-GFP fluorescence assayed by flow cytometry in HSCs from untreated littermate controls or mice treated with two doses of plpC weekly for 4 wk followed by 10-d recovery before analysis. At least two independent experiments were performed. **(E)** YTHDF2-GFP MFI of the data shown in D (*n* = 5 and *n* = 3 biological replicates, for treated and untreated, respectively). Data represent mean ± SEM; *, P < 0.05 (Mann–Whitney *U* test); at least two independent experiments were performed. **(F)** *Ythdf2*^fl/fl^;*Vav-iCre* (*Ythdf2*^CKO^) and control *Ythdf2*^fl/fl^ (*Ythdf2*^CTL^) mice were treated with two doses of plpC per week for 4 wk and allowed to recover for 10 d before analysis (Fig. 4 G). Furthermore, 200 LSK CD48^−^CD150^+^ CD45.2^+^ BM HSCs were sorted and transplanted into lethally irradiated 8–10-wk-old syngeneic CD45.1^+^/CD45.2^+^ recipient mice (*n* = 12 per genotype) together with 2 × 10^5^ CD45.1^+^ competitor BM cells (Fig. 4 H). **(G)** Total number of lineage^−^Sca-1^+^c-Kit^+^ LSK cells, HSCs (LSK CD34^−^CD135^−^CD150^+^CD48^−^), MPP1 (LSK CD34^+^CD135^−^CD150^+^CD48^−^), MPP2 (LSK CD34^+^CD135^−^CD150^+^CD48^+^), MPP3 (LSK CD34^+^CD135^−^CD150^−^CD48^+^), and MPP4 (LSK CD34^+^CD135^+^CD150^−^CD48^+^) cell populations in the BM of plpC-treated *Ythdf2*^CKO^ or *Ythdf2*^CTL^ mice. Data represent mean ± SEM; **, P < 0.01; ns, not significant (Mann–Whitney *U* test); at least two independent experiments were performed. **(H)** Percentage of CD45.2^+^ cells in the CD11b^+^Gr-1^−^ monocyte, CD11b^+^Gr-1^+^ granulocyte, CD19^+^ B cell, and CD4^+^ or CD8^+^ T cell, compartments of recipients transplanted with HSCs from plpC treated*Ythdf2*^CKO^ or *Ythdf2*^CTL^ mice 1 mo after transplantation (*n* = 12 and *n* = 13 recipients, respectively). Data represent mean ± SEM; ***, P < 0.001; ****, P < 0.0001 (Mann–Whitney *U* test); at least two independent experiments were performed.

Given that YTHDF2 expression is induced by stress in other contexts (Yu et al., 2019; Zhou et al., 2015) and is known to be up-regulated in immune cells during the IFN response (Rubio et al., 2018; Winkler et al., 2019), we hypothesized that YTHDF2 may itself be regulated by inflammatory stimuli in HSCs. To address this, we used *Ythdf2*^CTL^ mice harboring the GFP-YTHDF2 fusion protein, in which GFP analyzed by flow cytometry reports the expression of YTHDF2 protein. These mice were treated with two or eight doses of pIpC to induce the IFN-α response and model acute or chronic inflammation, respectively (Fig. 4 A). We found a significant increase in the expression of GFP-YTHDF2 protein in HSCs under both conditions (Fig. 4, B–E). Given these findings indicating that YTHDF2 is induced by inflammatory stimuli, we next examined how loss of YTHDF2 impacts the behavior of HSCs upon inflammation, by subjecting *Ythdf2*^CTL^ and *Ythdf2*^CKO^ mice to pIpC-induced chronic inflammation before allowing recovery (Fig. 4 F). As in mice under steady-state conditions (Paris et al., 2019; Fig. 1 I), primitive progenitor cell populations (MPP2–4) were expanded in pIpC-treated *Ythdf2*^CKO^ mice compared with their *Ythdf2*^CTL^ counterparts (Fig. 4 G). Strikingly, however, unlike during steady-state conditions in which *Ythdf2*-deficient HSCs and MPP-1 cells (also referred to as short-term HSCs) were expanded (Paris et al., 2019; Fig. 1 I), pIpC-treated *Ythdf2*^CKO^ and *Ythdf2*^CTL^ mice displayed comparable numbers of HSCs and MPP-1 cells (Fig. 4 G). Therefore, while *Ythdf2*-deficient HSCs (which display up-regulated proinflammatory pathways) undergo expansion under steady-state conditions, additional inflammatory stimuli abrogate this phenotype.

Finally, to investigate whether *Ythdf2* deficiency in HSCs had any impact on their short-term reconstitution potential upon pIpC administration, we competitively transplanted equal numbers of HSCs from pIpC-treated *Ythdf2*^CKO^ and *Ythdf2*^CTL^ mice into lethally irradiated recipients (Fig. 4 F). HSCs lacking *Ythdf2* displayed decreased short-term myeloid and B-lymphoid reconstitution capacity, indicating that inflammation temporarily weakened *Ythdf2*-deficient HSC activity (Fig. 4 H). Thus, our data indicate that YTHDF2 is induced by inflammation in HSCs and suggest that YTHDF2 may function to protect HSCs from excessive proinflammatory signals. The mechanisms through which YTHDF2 is induced by inflammation, and its precise involvement in protecting HSCs in this context, remain important open questions meriting further investigations.

Collectively, YTHDF2 expression is induced in HSCs by inflammatory stimuli and its deletion results in constitutive activation of proinflammatory pathways, consistent with myeloid bias. While YTHDF2 is not essential for long-term steady-state unperturbed hematopoiesis upon aging, *Ythdf2* deletion causes progressive HSC expansion and failure of HSCs to reconstitute multilineage hematopoiesis. Because the activation of inflammatory pathways in HSCs by infections or injury causes their proliferation and myeloid cell production, but in the longer term compromises HSC activity (Esplin et al., 2011; Essers et al., 2009; Pietras, 2017; Takizawa et al., 2017), HSCs must use mechanisms to counteract these signals and protect their long-term integrity. While several studies indicated that proinflammatory signals are suppressed in HSCs at different levels of gene expression, including cell signaling, gene transcription, and RNA editing (Hartner et al., 2009; King et al., 2011; Sato et al., 2009), the functional significance of dynamic RNA modifications in this important aspect of HSC biology remains poorly understood. We suggest that the m^6^A-dependent mRNA decay pathway is a novel mechanism through which HSCs suppress inflammation and sustain HSC integrity. Interrogation of up-regulated proinflammatory genes upon *Ythdf2* deficiency in genetic rescue approaches is warranted to further support this notion. Given our findings that YTHDF2 itself is induced by inflammation, we suggest that YTHDF2 may function in a negative feedback loop to prevent an excessive inflammatory response detrimental to HSC function. Thus, our work sets the stage for further investigations focusing on how YTHDF2 regulates HSC fates during inflammation upon acute and chronic infection. In closing, our data position YTHDF2 as an essential repressor of proinflammatory pathways in HSCs and highlight the key significance of m^6^A mRNA modification in long-term HSC maintenance upon aging.

## Materials and methods

### Mice

All mice were on the C57BL/6 background (backcrossed for ≥10 generations). *Ythdf2^fl/fl^*, *Vav-iCre*, and *Mx1-Cre* strains have been described previously (de Boer et al., 2003; Ivanova et al., 2017; Kühn et al., 1995). Congenic recipient mice were CD45.1^+^/CD45.2^+^. All experiments involving mice were performed under University of Edinburgh Veterinary oversight with UK Home Office authorization. All control mice were littermates, and treatment groups were randomized among littermates.

### Flow cytometry

BM and splenic cells were prepared and analyzed as described previously (Guitart et al., 2017; Guitart et al., 2013; Kranc et al., 2009; Mortensen et al., 2011; Paris et al., 2019; Vukovic et al., 2016). Hematopoietic stem and progenitor cell staining began with incubation with Fc block followed by biotin-conjugated anti-Lineage marker antibodies (anti-CD4, anti-CD5, anti-CD8a, anti-CD11b, anti-B220, anti-Gr-1, and anti-Ter119), APC-conjugated anti-c-Kit, APC-Cy7–conjugated anti-Sca-1, PE-conjugated anti-CD48, and PE-Cy7–conjugated anti-CD150 antibodies. Biotin-conjugated lineage markers were then stained with PerCP-conjugated streptavidin. Staining of MPP1–4 populations was performed as above for hematopoietic stem and progenitor cells; however, BV711- instead of APC-conjugated anti-c-Kit was used and cells were also stained with APC-conjugated anti-Flt3– and BV421-conjugated anti-CD34 antibodies.

For PB and differentiated cell analysis, cells were stained with PerCP-conjugated anti-B220, APC-Cy7–conjugated anti-CD19, Pacific Blue–conjugated anti-CD11b, PE-Cy7–conjugated anti-Gr-1, APC-conjugated anti-CD8, and PE-conjugated anti-CD4 antibodies. To distinguish CD45.2 chimerism in transplantation experiments, BV711-conjugated anti-CD45.1 and Pacific Blue–conjugated anti-CD45.2 antibodies were used. For hematopoietic stem and progenitor cell staining in transplanted mice, APC-conjugated anti-c-Kit and APC-Cy7-conjugated anti-Sca-1 were used; the remainder of the staining was as described above. For analyses of differentiated cells and PB in transplanted mice, cell suspensions were stained with BV711-conjugated anti-CD45.1, Pacific Blue–conjugated anti-CD45.2, APC-conjugated anti-CD11b, PE-Cy7–conjugated anti-Gr-1, PE-conjugated anti-CD4 and -CD8a, and APC-Cy7–conjugated anti-CD19. Flow cytometry analyses were performed using a LSRFortessa (BD Biosciences). Cell sorting was performed on a FACSAria Fusion (BD Biosciences).

### Intracellular staining and flow cytometry analysis

BM and splenic cells were prepared and analyzed as described previously (Guitart et al., 2017; Guitart et al., 2013; Kranc et al., 2009; Mortensen et al., 2011; Paris et al., 2019; Vukovic et al., 2016) followed by c-Kit enrichment (described below). Cells were incubated with Fc block and stained with biotin-conjugated anti-Lineage marker antibodies (anti-CD4, anti-CD5, anti-CD8a, anti-CD11b, anti-B220, anti-Gr-1, and anti-Ter119), APC-Cy7–conjugated anti-c-Kit, and Pacific Blue–conjugated anti-Sca-1. Biotin-conjugated Lineage markers were then stained with PerCP-conjugated Streptavidin. Cells were then fixed and permeabilized using Phosflow Lyse/Fix, Phosflow Perm Buffer III, and stain buffer (all from BD Biosciences) according to the manufacturer’s instructions. After processing, cells were stained with AF647-conjugated anti-pStat1 or anti-pStat3.

### Syngeneic transplantation assays

Transplant recipient CD45.1^+^/CD45.2^+^ mice were lethally irradiated with two doses of 5.5 Gy administered ≥4 h apart at an average rate of 0.58 Gy/min using a cesium-137 GammaCell 40 irradiator. For primary transplantations, irradiated recipient CD45.1^+^/CD45.2^+^ mice were intravenously injected with 200 LSK CD48^−^CD150^+^ HSCs and 200,000 support CD45.1^+^ unfractionated BM cells. For secondary transplantations, recipient CD45.1^+^/CD45.2^+^ mice were intravenously injected with 2,000 CD45.2^+^ LSK cells or 100,000 c-Kit–enriched BM cells from primary recipients and 200,000 support CD45.1^+^ unfractionated BM cells. All recipient mice were culled and analyzed 16–20 wk after transplantation. A minimum of two independent donor mice were used for all transplantations.

### pIpC administration

For gene deletion via activation of the *Mx1-Cre* promotor, mice were injected i.p. every other day with 300 µg pIpC (GE Healthcare) for a total of six doses over 11 d, as previously described (Guitart et al., 2017; Guitart et al., 2013; Kranc et al., 2009; Paris et al., 2019), and gene deletion was assayed by YTHDF2-GFP fusion protein fluorescence. To test the effect of innate immune activation, mice were injected i.p. with 300 µg pIpC (GE Healthcare) as described per experiment.

### CD117 (c-Kit) enrichment

Enrichment for cells expressing CD117 (c-Kit) was performed using CD117 MicroBeads and LS columns from Miltenyi Biotec, according to the manufacturer’s instructions.

### Histology

Tibias and femurs were dissected and cleaned before fixation in 4% paraformaldehyde for 24 h. Fixed bones were decalcified, embedded in paraffin, and sectioned. Sections were stained with H&E and mounted. Whole sections were imaged using a Vectra Polaris slide scanner, and representative images were taken at 10× and 20× magnification.

### RNA-seq

100 HSCs per sample were sorted into 0.4% Triton X-100, RNase inhibitor, 10 mM dNTPs, and 10 µM Oligo dT before RNA-seq by SMART-Seq2 in the Single Cell Genomics Facility at the Medical Research Council Weatherall Institute of Molecular Medicine (University of Oxford). Alignments to the GRCm38 mouse genome were performed using HISAT2 (Kim et al., 2015), and further analysis was performed in R. Counts were assigned to genes using the Rsubread package, and quality control analysis of the samples was performed with the SingleCellExperiment and scater packages. Differentially expressed genes were identified using likelihood ratio tests computed by the edgeR package. The RNA-seq dataset was deposited in GEO (accession no. GSE142019).

### m^6^A meRIP-seq

m^6^A meRIP-seq library preparation was performed as previously described (Lin et al., 2016) from *Ythdf2*^CTL^ c-Kit–enriched cells. Reads from two biological replicates were aligned to the mouse GRCm38 genome using HISAT2 (Kim et al., 2015), and peaks were called using MACS2, version 2.1.1 with the nomodel flag (Zhang et al., 2008). To analyze the distribution of m^6^A-associated reads along the transcripts, treat_pileup bedgraph file outputs from MACS2 were converted to bigWig format and used as input for the computeMatrix function of the deepTools package, version 2.5.5 (Ramírez et al., 2014). Motif enrichment was done using Homer, version 4.10, selecting a motif length of six nucleotides. Background regions were generated by shuffling peaks along the transcriptome using the shuffleBed tool from the bedtools suite, version 2.28 (Quinlan and Hall, 2010). The GVIZ bioconductor package was used for peak visualization (Hahne and Ivanek, 2016). m^6^A meRIP-seq dataset was deposited in GEO (accession no. GSE142020).

### GSEA and IPA

GSEA was performed using GSEA software version 3.0 with 1,000 permutations and default parameters. Gene differential expression, computed by the edgeR package in R, was ranked by moderated *t* statistics, which takes into account variability between genes in the ranking. Ranked genes were compared with gene lists in the Hallmark subset of the MSigDB database, version 7.0. IPA was performed using the Core Analysis Function offered by Qiagen’s Ingenuity Pathway Analysis software (Krämer et al., 2014). The interrogated RNA-seq and mass spectrometry datasets were filtered for adjusted P values of differential expression (FDR < 0.05), and the threshold for significant activation or inhibition was defined by an absolute Z-score value >2.

### SLAM-seq analysis

The SLAM-seq dataset (Paris et al., 2019) was analyzed using the SlamDunk pipeline (Herzog et al., 2017).

### Mass spectroscopy analysis

Immortalized *Ythdf2*^CKO^ and *Ythdf2*^CTL^ c-Kit–enriched cells (Paris et al., 2019) were pelleted, lysed, and processed according to (Wiśniewski and Rakus, 2014). Tryptic peptides were analyzed on a QExactive+ mass spectrometer connected to an Ultimate Ultra3000 chromatography system packed with 1.8 µm uChrom and separated by an acetonitrile gradient. Mass spectra were analyzed using MaxQuant against the Uniprot mouse database proteome, ID UP000000589.

### Quantification and statistical analysis

Statistical analyses were performed using Prism 6 software (GraphPad Software). P values were calculated using a two-tailed Mann–Whitney *U* test unless stated otherwise.

### Online supplemental material

Fig. S1 shows the frequency of B cells, T cells, granulocytes, and monocytes within the CD45.2^+^ donor-derived population of PB, BM, and spleen from transplant recipients shown in Figs. 1 B; and 3 F, I, and K. Also shown are GSEA plots from RNA-seq of *Ythdf2*^CKO^ and *Ythdf2*^CTL^ HSCs from Fig. 1 F. Fig. S2 shows IPA of RNA-seq and mass spectrometry from *Ythdf2*^CKO^ and *Ythdf2*^CTL^ cells and mRNA decay curves for transcripts of interest within *Ythdf2*^CKO^ and *Ythdf2*^CTL^ c-Kit^+^ cells. Fig. S3 shows data to support our conclusions in aged mice (YTHDF2-GFP mean fluorescence is not significantly altered with age; aged mice have normal BM cellularity and histology); percentage of monocytes, granulocytes, CD4^+^ T cells, CD8^+^ T cells, B cells, and GFP^+^ cells in PB from mice 1 yr after *Ythdf2* deletion; and total blood counts, numbers of differentiated cells, and numbers of HSCs for mice 1 yr after *Ythdf2* deletion.
